# Association between Intra-Arrest Blood Glucose Level and Outcomes of Resuscitation at the Emergency Department: A Retrospective Study

**DOI:** 10.3390/jcm11113067

**Published:** 2022-05-29

**Authors:** Wachira Wongtanasarasin, Nat Ungrungseesopon, Phichayut Phinyo

**Affiliations:** 1 Department of Emergency Medicine, Faculty of Medicine, Chiang Mai University, Chiang Mai 50200, Thailand; nat.u@cmu.ac.th; 2 Department of Emergency Medicine, UC Davis School of Medicine, Sacramento, CA 95817, USA; 3 Department of Family Medicine, Faculty of Medicine, Chiang Mai University, Chiang Mai 50200, Thailand; phichayutphinyo@gmail.com; 4 Center for Clinical Epidemiology and Clinical Statistics, Faculty of Medicine, Chiang Mai University, Chiang Mai 50200, Thailand

**Keywords:** cardiac arrest, blood glucose, emergency department, survival, clinical outcome

## Abstract

Since current cardiac arrest guidelines do not address the benefit of blood glucose measurement, the ideal ranges and target of blood glucose (BG) levels during cardiac arrest to achieve a better result are warranted. We intended to investigate the associations between intra-arrest BG levels and outcomes of cardiac arrest resuscitation at the emergency department (ED). We conducted a retrospective observational study at a single university hospital. Cardiac arrest patients at the ED between 2017 and 2020 were included. Multivariable logistic regression analysis was performed to examine the associations between intra-arrest BG levels and clinical outcomes. We categorized intra-arrest BG into five groups: <70 mg/dL, 70–99 mg/dL, 100–180 mg/dL, 181–250 mg/dL, and >250 mg/dL. Eight hundred and nineteen patients experienced ED cardiac arrest during the study period. Of all, 385 intra-arrest BG measurements were included in the data analysis. The mean age was 60.4 years. The mean intra-arrest BG level was 171.1 mg/dL, with 64 (16.6%) patients who had intra-arrest BG level below 70 mg/dL and 73 (19.0%) patients who had intra-arrest BG level more than 250 mg/dL. Markedly low (<70 mg/dL) and low (70–99 mg/dL) intra-arrest BG levels were significantly associated with a lower chance of return of spontaneous circulation (ROSC, OR 0.36, 95% CI 0.14–0.99, *p* = 0.05 and OR 0.33, 95% CI 0.12–0.93, *p* = 0.04, respectively). For patients who experienced cardiac arrest at the ED, an intra-arrest BG level of less than 100 was inversely correlated with sustained ROSC. Although we could not draw a causal relationship between variables concerning this study design, normalizing intra-arrest BG was shown to result in good clinical outcomes.

## 1. Introduction

Cardiac arrest is recognized as one of the most important causes of mortality and disability [1]. In the United States, there are around 40,000 patients who are diagnosed with sudden cardiac arrest and undergo cardiopulmonary resuscitation (CPR) in emergency departments (EDs) each year [2]. Cardiopulmonary resuscitation is a lifesaving procedure that attempts to restore adequate cardiopulmonary function and tissue perfusion [3]. During CPR, the 2020 American Heart Association Guidelines for Advanced Cardiac Life Support (ACLS) has suggested that reversible causes of arrest should be identified and managed immediately [4].

Blood glucose (BG) is one of the significant parameters to be assessed during cardiac arrest as it plays a significant role in many cell metabolisms [5]. It is well established that a hypoglycemic state during cardiac arrest is related to poor outcomes [6]. Hypoglycemia has been removed from the reversible causes of cardiac arrest since the 2010 ACLS guidelines. In addition, measuring BG and acute management of hypoglycemia during CPR was not discussed in a subsequent edition [7]. On the other hand, a previous case report documented a sudden return of spontaneous circulation (ROSC) after administration of dextrose for hypoglycemia (10.8 mg/dL) [8]. However, a study by Wang and colleagues demonstrated that dextrose administration did not pose any benefits, including survival to hospital discharge and favorable neurological outcomes, during cardiac arrest [9]. Unlike hypoglycemia, hyperglycemia during cardiac arrest was not well established to be related to a higher chance of return of spontaneous circulation (ROSC) or neurological outcomes [8]. Apart from the intra-arrest phase, the BG level also plays a powerful role in post-resuscitation care [10]. Previous articles illustrated that post-resuscitation blood glucose levels were associated with neurological outcomes. In addition, hyperglycemia has been found to be associated with poor neurological recovery [6,11,12,13].

Regardless of this knowledge, the optimal range for this point is not well-established, and there is currently no strong consensus on this point. Moreover, the benefit of intra-arrest BG normalization during CPR is not well-documented. As a result, this study aims to investigate a relationship between BG level and resuscitation outcomes, including the probability of ROSC, survival to hospital admission, survival to hospital discharge, and favorable neurological outcome at hospital discharge [14,15].

## 2. Materials and Methods

### 2.1. Study Design and Setting

This study was conducted following the recommendations to strengthen the reporting of observational studies in epidemiology (STROBE) statement [16]. We conducted a retrospective cohort study in the ED at a university tertiary hospital, Maharaj Nakorn Chiang Mai Hospital (MNCMH). Maharaj Nakorn Chiang Mai has approximately 30,000 annual ED visits and consists of 1500 patient beds, 151 intensive care units (ICU) and sub-ICU beds, and 28 operating rooms. Our study was performed following the Declaration of Helsinki statements. The study was approved by the Institutional Review Board (Approval No. 7477/2020) before data collection.

### 2.2. Participants

Patients who experienced cardiac arrest at ED at MNCMH between January 2017 and December 2020 were screened for eligibility. Inclusion criteria were: (1) aged ≥18 years, (2) recorded absence of pulse, which required CPR at the ED, (3) no evidence or documentation of a do-not-attempt-resuscitation (DNAR) order before cardiac arrest, and (4) measurement of BG level during CPR. If a cardiac arrest occurred more than once in a single patient during an ED stay, only the first event was recorded and included in this study. Patients who had an out-of-hospital cardiac arrest (OHCA) without prehospital ROSC and were transferred to ED were excluded from this study.

### 2.3. Data Collection and Outcome Measures

We extracted clinical information from our cardiac arrest registry. Paramedics and nurses who had experience working in the ED for at least two years were abstractors in our study. Trained abstractors reviewed participants’ hospital medical records and entered them into the REDcap platform (Vanderbilt University, Nashville, TN) simultaneously. Specifically, we collected data on age, sex, time of arrival, cardiac arrest mechanism, initial presenting rhythm, medications administered during cardiac arrest, including adrenaline, amiodarone, lidocaine, dextrose, CPR duration, and the first intra-arrest BG obtained during CPR. Intra-arrest BG level was measured using various point-of-care devices with varying degrees of accuracy and usability. In most cases, BG has been measured from blood collected shortly after intravenous access before any medication is administered during CPR. In this study, BG levels were categorized in 5 groups: <70 mg/dL (3.89 mmol/L), 70–99 mg/dL (3.89–5.49 mmol/L), 100–180 mg/dL (5.55–9.99 mmol/L), 181–250 mg/dL (10.05–13.88 mmol/L), and >250 mg/dL (13.88 mmol/L). Hypoglycemia and hyperglycemia limits are not regularly specified and vary greatly in the literature. The threshold of 70 mg/dL (3.89 mmol/L) is often used to define hypoglycemia [17]. In contrast, the threshold of 250 mg/dL (13.88 mmol/L) is one of the diagnostic criteria for diabetic ketoacidosis, a life-threatening complication of hyperglycemia [18]. However, prior studies on the effects of hypoglycemia on cardiac arrests used a wide range of cut-off values ranging from 100–150 mg/dL [6,9,14,19]. According to the American Diabetes Association (ADA) and American Association of Clinical Endocrinologists (AACE) task force on inpatient glycemic control [20], a glucose level between 140 and 180 mg/dL has been recommended for the ICU patients [21]. Moreover, a glucose level between 100 and 180 mg/dL has been recommended for selected ICU patients (i.e., centers with extensive experience and appropriate nursing support, cardiac surgical patients) [21].

The primary outcome was sustained ROSC at the ED. Sustained ROSC was defined as ROSC lasting at least 20 min without the need to resume chest compressions. Secondary outcomes included survival to hospital admission, survival to hospital discharge, and favorable neurological outcome at hospital discharge. Favorable neurological status was defined as having the Cerebral Performance Category (CPC) score of 1 or 2. The CPC score was derived retrospectively by reviewing the medical records of each patient.

### 2.4. Statistical Analysis

Stata 16 (StataCorp, College Station, TX, USA) was used to conduct all data analyses. Categorical variables were described using numbers and proportions and analyzed using the chi-squared test’s crosstabs. Continuous variables were described using means and standard deviations and analyzed using one-way variance analysis (ANOVA). Statistical significance was defined as a two-tailed *p*-value of <0.05. The odds ratio (OR) was chosen as the endpoint of measurement, and multivariable logistic regression analyses were used to investigate the relationships between the independent factors and the outcomes. The regression model considered all available independent variables, regardless of whether they were significant in univariate analysis. We also presented a figure to demonstrate intra-arrest BG levels and ORs for ROSC at the ED for the primary outcome.

## 3. Results

### 3.1. Participants

**Figure 1:**
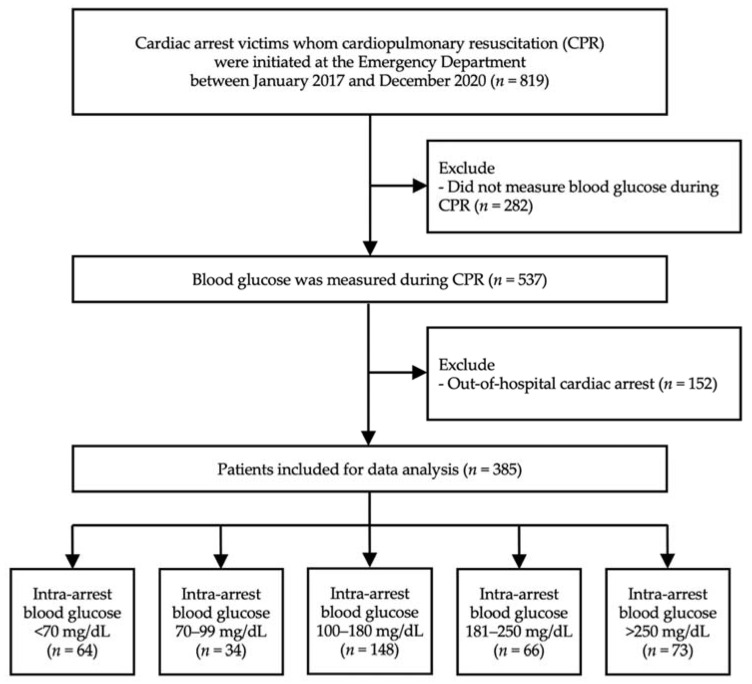
Study flow.

During the study period, 819 patients at MNCMH received CPR at the ED. Finally, the remaining 385 patients were included in the data analysis (Figure 1). Table 1 demonstrates the baseline demographics, clinical characteristics, features, and interventions for all patients in the cohort. The mean age of the participants was 60.4 years. Most of them were male (61.3%). A total of 71 (18.4%) cardiac arrest events were traumatic mechanisms. The average CPR duration was 21.3 min without a difference observed among groups (*p* = 0.30). The mean intra-arrest BG level was 171.1 mg/dL (9.51 mmol/L), with 64 (16.6%) patients who had intra-arrest BG below 70 mg/dL. Intravenous dextrose (50% dextrose in water) was injected in 69 (17.9%) cardiac arrest patients.

### 3.2. Outcomes

Approximately half of the participants (48.8%) achieved sustained ROSC. Of these, 120 (31.3%) patients survived to hospital admission, 43 (11.4%) survived to hospital discharge, and only 13 (3.5%) had a favorable neurological status at discharge. Sustained ROSC was higher in normal intra-arrest BG group (*p* < 0.001), compared with the other groups. Patients with intra-arrest BG between 100 and 180 mg/dL had the highest sustained ROSC, survival rate to hospital admission, and survival rate to hospital discharge (61.5%, 40.1%, and 13.3%, respectively). Table 2 demonstrates all clinical outcomes of the study population.

**Figure 2:**
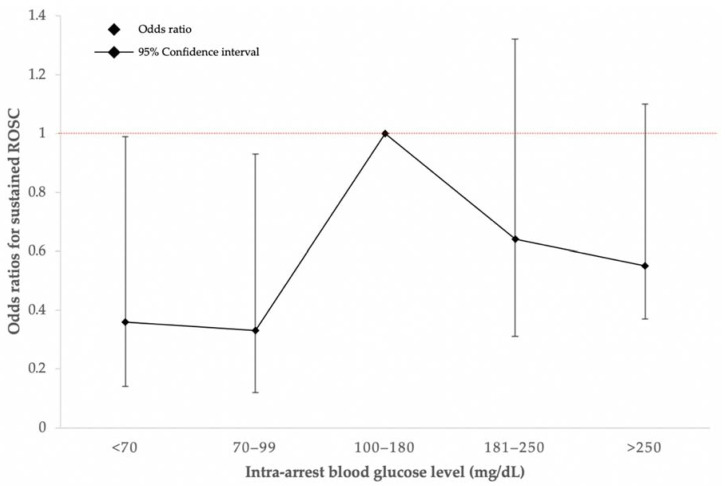
Intra-arrest blood glucose level and the chance of having sustained ROSC. ROSC, return of spontaneous circulation.

As shown in Table 3, BG levels of <70 mg/dL and 70–99 mg/dL were significantly associated with a lower sustained ROSC (adjusted OR, aOR 0.36, 95% CI: 0.14–0.99, *p* = 0.05, aOR 0.33, 95% CI: 0.12-0.93, *p* = 0.04, respectively). Figure 2 illustrates the trend of each intra-arrest BG group and the chances of having sustained ROSC.

## 4. Discussion

Our study found that hypoglycemia, defined by intra-arrest BG of less than 100 mg/dL, was associated with lower rates of sustained ROSC. Hypoglycemia was considered one of the reversible causes of cardiac arrest in 2005 guidelines [22]. Later, it was removed in the subsequent guidelines (2010 [23], 2015 [24], and 2020 [4]). Moreover, the 2020 guidelines [4] did not address whether BG levels should be assessed during CPR or if intra-arrest hypoglycemia or hyperglycemia should be treated. As shown in our results, it is demonstrated that optimal intra-arrest BG levels were correlated with good clinical outcomes. Several studies found that disturbance of BG regulation after resuscitation from cardiac arrest is common [12,25,26]. Recently, a large cohort in Taiwan found that intra-arrest BG less than 150 mg/dL was associated with worse neurological outcomes [9]. Together with our findings, intra-arrest BG could be a marker of resuscitation prognosis.

Interestingly, our study found that the proportion of traumatic cardiac arrest was fairly high (18.4%), which is in contrasted with previous reports [27,28]. Our hospital represents a tertiary university hospital where complicated and sophisticated patients were referred for definite investigations and management, including severe traumatic patients. Traumatic cardiac arrest patients may require different approaches and specific management. However, international guidelines still recommend treating traumatic cardiac arrest following the ACLS guidelines organized by the American Heart Association (AHA)/European Resuscitation Council (ESC) [4]. In addition, the mechanism of cardiac arrest was adjusted in the multivariable analysis to reduce any confounding bias arising from this variable.

In animal studies, it has been documented that severe hypoglycemia (10–15 mg/dL) might lead to fatal ventricular arrhythmias (i.e., premature ventricular contraction, ventricular tachycardia, atrioventricular block, etc.) [29]. Interestingly, interventions to normalize intra-arrest BG levels are still debatable. Despite showing in a case report [8] that the administration of dextrose during hypoglycemic cardiac arrest resulted in a rapid ROSC, Wang et al. reported that dextrose administration was not correlated with improved outcomes of in-hospital cardiac arrest patients with intra-arrest BG <150 mg/dL [9]. On the other hand, hyperglycemia is also common during CPR [25]. Cardiac arrest, recognized as major stress, causes severe metabolic derangements and global ischemia/reperfusion (IR) injury [25]. The connections between cardiac IR injury, fatal ventricular arrhythmias, and cardiac arrest have been well established across preclinical and clinical studies [30,31]. These links also affect the endocrine glands, including the pancreas, and possibly lead to reduced hormonal synthesis, resulting in lower insulin and glucagon-like peptide 1 (GLP-1) levels. Several studies presented a strong argument that high BG levels after ROSC positively correlate with increased mortality and poor neurological outcomes [6,11,12]. However, there is still limited evidence regarding drugs to reduce BG levels during resuscitation.

This study has some limitations that need to be documented. Because of the retrospective observational design of this study, we could not draw a cause–effect relationship between intra-arrest blood glucose levels and outcomes. Several factors influenced intra-arrest blood glucose levels, e.g., pre-arrest events and medications, past medical history of diabetes, diet, and nutritional status. Second, since data in this study were obtained from only one university hospital, it might not be directly applied in other settings. Furthermore, this study did not include cardiac arrest patients whose intra-arrest BG was not measured, which may result in selection bias. In addition, some factors could influence blood glucose levels. However, we believe that these links may not eventually lead the patient to a cardiac arrest event. Practically, most clinicians would not know the exact causes of cardiac arrest at the moment of resuscitation. A future study searching for any causes of hypo/hyperglycemia before the cardiac arrest event occurs could address this issue. Moreover, the effects of unmeasured confounders may have affected the results despite using multivariable analysis to compensate for the impact of measured confounding factors. To clarify, pre-arrest events that resulted in relatively low or high BG levels, pre-arrest comorbidities, diabetes status, duration of cardiac arrest since patients arrived at the ED, and the reasons why intra-arrest BG levels were taken may be relevant confounding factors and could not be validated retrospectively. Consequently, a prospective, well-designed study needs to be conducted to solve these limitations.

## 5. Conclusions

In ED cardiac arrests, initial glucose measurements of <100 mg/dL was associated with poorer odds of sustained ROSC. Although we could not draw a conclusion concerning this study design, optimal intra-arrest BG levels should be considered when resuscitating cardiac arrest patients. However, interventions that have been proven successful in achieving ROSC, e.g., high-quality CPR and defibrillation when indicated, should be mainly focused on. A future prospective study addressing this cause–effect relationship is warranted.

## Figures and Tables

**Table 1:** Baseline demographics, clinical characteristics, features, and interventions of the study population.

Variables	All Patients (*n* = 385)	Intra-Arrest BG < 70 mg/dL (*n* = 64)	Intra-Arrest BG 70–99 mg/dL (*n* = 34)	Intra-Arrest BG 100–180 mg/dL (*n* = 148)	Intra-Arrest BG 181–250 mg/dL (*n* = 66)	Intra-Arrest BG > 250 mg/dL (*n* = 73)	*p*-Value
Age, year (SD)	60.4 (20.7)	61.5 (20.7)	53.0 (21.1)	61.6 (21.7)	63.7 (21.2)	57.2 (17.2)	0.27
Male, *n* (%)	236 (61.3)	32 (50.0)	23 (67.7)	94 (63.5)	42 (63.6)	45 (61.6)	0.35
Time of arrival at ED, *n* (%)							0.26
8.01–16.00	139 (36.1)	29 (45.3)	15 (44.1)	54 (36.5)	17 (25.8)	24 (32.9)	
16.01–24.00	126 (32.7)	15 (23.4)	11 (32.4)	49 (33.1)	29 (43.9)	22 (30.1)	
24.00–8.00	120 (31.2)	20 (31.3)	8 (23.5)	45 (30.4)	20 (30.3)	27 (37.0)	
Traumatic mechanism, *n* (%)	71 (18.4)	8 (12.5)	9 (26.5)	27 (18.2)	13 (19.7)	14 (19.2)	0.55
Initial shockable rhythm, *n* (%)	58 (15.1)	6 (9.4)	5 (14.7)	22 (14.9)	13 (19.7)	12 (16.4)	0.59
Adrenaline given during cardiac arrest, *n* (%)	378 (98.2)	61 (95.3)	34 (100.0)	145 (98.0)	66 (100.0)	72 (98.6)	0.30
Amiodarone given during cardiac arrest, *n* (%)	46 (12.0)	1 (1.6)	3 (8.8)	14 (9.5)	16 (24.2)	12 (16.4)	0.001
Lidocaine given during cardiac arrest, *n* (%)	27 (7.0)	0 (0)	1 (2.9)	11 (7.4)	9 (13.6)	6 (8.2)	0.04
Dextrose given during cardiac arrest, *n* (%)	69 (17.9)	51 (79.7)	5 (14.7)	8 (5.4)	3 (4.6)	2 (2.7)	<0.001
CPR duration, min (SD)	21.0 (12.9)	21.9 (10.8)	24.6 (14.6)	18.7 (12.9)	23.2 (13.2)	21.0 (13.0)	0.30
Intra-arrest blood glucose level, mg/dL (SD)	171.1 (111.4)	41.0 (17.9)	85.6 (8.7)	138.7 (22.8)	213.3 (19.2)	352.7 (93.8)	<0.001

Abbreviations: BG, blood glucose; CPR, cardiopulmonary resuscitation; ED, emergency department; SD, standard deviation.

**Table 2:** Clinical endpoints of the study population.

Endpoints	All patients (*n* = 385)	Intra-arrest BG < 70 mg/dL (*n* = 64)	Intra-Arrest BG 70–99 mg/dL (*n* = 34)	Intra-Arrest BG 100–180 mg/dL (*n* = 148)	Intra-Arrest BG 181–250 mg/dL (*n* = 66)	Intra-Arrest BG > 250 mg/dL (*n* = 73)	*p*-Value
Sustained ROSC, *n* (%)	188 (48.8)	20 (31.3)	12 (35.3)	91 (61.5)	28 (42.4)	37 (50.7)	<0.001
Survival to hospital admission, *n* (%)	120 (31.3)	11 (17.2)	8 (23.5)	59 (40.1)	20 (30.3)	22 (30.1)	0.02
Survival to hospital discharge, *n* (%)	43 (11.4)	3 (4.8)	4 (11.8)	19 (13.3)	8 (12.3)	9 (12.5)	0.49
Favorable neurological outcome at discharge, *n* (%)	13 (3.5)	1 (1.6)	1 (3.0)	5 (3.6)	1 (1.6)	5 (7.1)	0.39

Abbreviations: BG, blood glucose; ROSC, return of spontaneous circulation.

**Table 3:** Multiple logistic regression model with each outcome as the dependent variable stratified by intra-arrest blood glucose.

Outcomes	Odds Ratios	95% Confidence Intervals	*p*-Value
Sustained return of spontaneous circulation			
<70 mg/dL	0.36	0.14–0.99	0.05
70–99 mg/dL	0.33	0.12–0.93	0.04
100–180 mg/dL	1	Reference	-
181–250 mg/dL	0.64	0.31–1.32	0.23
>250 mg/dL	0.55	0.37–1.10	0.09
Survival to hospital admission			
<70 mg/dL	0.32	0.10–1.03	0.06
70–99 mg/dL	0.49	0.17–1.48	0.21
100–180 mg/dL	1	Reference	-
181–250 mg/dL	1.04	0.49–2.19	0.92
>250 mg/dL	0.58	0.28–1.18	0.13
Survival to hospital discharge			
<70 mg/dL	0.67	0.12–3.84	0.66
70–99 mg/dL	0.91	0.23–3.69	0.90
100–180 mg/dL	1	Reference	-
181–250 mg/dL	1.67	0.61–4.57	0.32
>250 mg/dL	0.89	0.34–2.33	0.82
Favorable neurological outcome at discharge			
<70 mg/dL	1.36	0.08–23.47	0.83
70–99 mg/dL	1.16	0.09–15.53	0.91
100–180 mg/dL	1	Reference	-
181–250 mg/dL	1.03	0.08–12.70	0.98
>250 mg/dL	1.81	0.33–10.01	0.50

## Data Availability

Not applicable.
